# Differential gene expression of tumor-infiltrating CD33^+^ myeloid cells in advanced- versus early-stage colorectal cancer

**DOI:** 10.1007/s00262-020-02727-0

**Published:** 2020-09-30

**Authors:** Salman M. Toor, Rowaida Z. Taha, Varun Sasidharan Nair, Reem Saleh, Khaled Murshed, Mohamed Abu Nada, Eyad Elkord

**Affiliations:** 1 Cancer Research Center, Qatar Biomedical Research Institute (QBRI), Hamad Bin Khalifa University (HBKU), Qatar Foundation (QF), P.O. Box: 34110, Doha, Qatar; 2 Department of Pathology, Hamad Medical Corporation, Doha, Qatar; 3 Department of Surgery, Hamad Medical Corporation, Doha, Qatar

**Keywords:** Colorectal cancer, Tumor microenvironment, Myeloid cells, Transcriptomics

## Abstract

Colorectal cancer (CRC) has high mortality rates, especially in patients with advanced disease stages, who often do not respond to therapy. The cellular components of the tumor microenvironment are essentially responsible for dictating disease progression and response to therapy. Expansion of different myeloid cell subsets in CRC tumors has been reported previously. However, tumor-infiltrating myeloid cells have both pro- and anti-tumor roles in disease progression. In this study, we performed transcriptomic profiling of cells of myeloid lineage (CD33^+^) from bulk CRC tumors at varying disease stages. We identified differentially expressed genes and pathways between CRC patients with advanced stage and early stages. We found that pro-angiogenic and hypoxia-related genes were upregulated, while genes related to immune and inflammatory responses were downregulated in CD33^+^ myeloid cells from patients with advanced stages, implying that immune cell recruitment and activation could be compromised in advanced disease stages. Moreover, we identified a unique “poor prognosis CD33^+^ gene signature” by aligning top upregulated and downregulated genes in tumor-infiltrating myeloid cells from our analyses with data from The Cancer Genome Atlas. Our results showed that this gene signature is an independent prognostic indicator for disease-specific survival in CRC patients, potentially reflecting its clinical importance.

**Electronic supplementary material:**

The online version of this article (10.1007/s00262-020-02727-0) contains supplementary material, which is available to authorized users.

## Introduction

Colorectal cancer (CRC) is the third leading cause of all cancer-related deaths in the world, and its incidence is about threefold higher in developed countries compared to rest of the world [1]. CRC comprises a heterogeneous group of diseases, resulting from the combined effects of various mutations and mutagens [2]. Patients with advanced-stage/metastatic CRC mainly receive palliative therapies for disease management, and majority of therapies prove ineffective. Drug resistance and tumor recurrence are common in these patients, leading to the devastating mortality rates of CRC [3]. Emerging evidences have shown that investigating the transcriptome of CRC tumors can provide better insights into tumor characteristics and assist to predict response to treatment [4]. Additionally, the immune cellular components of the tumor microenvironment (TME) are largely responsible for response to therapies [5]. Therefore, it is vital to explore their molecular characteristics, which contribute to various mechanisms/pathways to aid tumor progression.

Myeloid cells can be present as the most abundant cell type in the TME and affect patient outcomes in many cancer types [6]. Cells of myeloid lineage reach terminal differentiation and are trafficked to the TME in response to specific colony-stimulating factors and soluble cytokines. Myeloid cells in the TME carry out both pro- and anti-tumor roles. For instance, monocytes/macrophages/monocyte-derived dendritic cells (Mo-DCs) in the TME may be involved in phagocytosis, secretion of tumoricidal mediators, promotion of angiogenesis, remodeling of the extracellular matrix and recruitment of lymphocytes [7]. While, tumor-infiltrating myeloid antigen-presenting cells are involved in T cell activation, immunosuppressive myeloid cells, such as tumor-associated macrophages (TAMs) and myeloid-derived suppressive cells (MDSCs), hamper anti-tumor therapies and assist disease progression [8]. MDSCs are immunosuppressive cells that have been associated with worse prognosis in various solid tumors and malignancies [9, 10]. MDSCs are recruited to the TME in response to various factors produced by the tumor stroma (e.g., pro-inflammatory cytokines, HMGB1), prostaglandin E2 (PGE2) and cyclooxygenase 2 (COX2) [9]. Numerous studies have reported the expansion of myeloid suppressor cells in the TME of CRC patients and their associations with disease progression [11–13]. However, uncovering pathways and mechanisms employed by myeloid cells in the TME, in addition to their role in response to virtually all therapeutic modalities, are largely unknown.

In this study, we sorted tumor-infiltrating myeloid cells from bulk tumors of CRC patients with varying disease stages, and performed comprehensive transcriptomic analyses to reveal their possible roles in disease progression. Our data highlighted the various unique and overlapping pathways shared between tumor-infiltrating myeloid cells of patients with varying disease stages. Aligning our data with The Cancer Genome Atlas (TCGA) for colon and rectal cancer enabled us to identify a distinct gene signature of tumor-infiltrating myeloid cells in CRC patients that serves as an independent prognostic indicator for disease-specific survival, referred to as “the poor prognosis CD33^+^ gene signature”. Collectively, our findings can have important implications on the current knowledge of myeloid cell contributions to disease progression in CRC patients.

## Materials and methods

### Sample collection and storage

Tumor tissues (TT) were obtained from treatment-naïve CRC patients (*n* = 13), who underwent surgery at Hamad Medical Corporation, Doha, Qatar. Table 1 shows the clinical and pathological features of all participating patients. All patients included in this study provided written informed consent prior to sample collection. This study was executed under ethical approvals from Hamad Medical Corporation, Doha, Qatar (protocol no. MRC-02-18-012) and Qatar Biomedical Research Institute, Doha, Qatar (protocol no. 2017-006). All experiments were executed in accordance with applicable guidelines and regulations.

**Table 1:** Characteristic features of study populations

	CRC patients
Number	13
Age (median)	64 (23–78)^†^
Gender (Male:Female)	6:7
TNM stage	
I	5
II	3
III	1
IV	4
Histological grade	
G2 Moderately differentiated	12
G3 Poorly differentiated	1

*CRC* colorectal cancer

^†^Median age

Tissue specimens were cut into small pieces and frozen in 1 ml of freezing medium [40% RPMI-1640 medium (Life Technologies, New York, USA), 50% fetal calf serum (FCS; Hyclone, GE Healthcare Life Sciences, Utah, USA) and 10% dimethylsulfoxide (DMSO; Sigma-Aldrich, Missouri, USA) and stored in liquid nitrogen to be used in batches for subsequent analyses, as previously described [14].

### Dissociation of tissues

Cell suspensions were prepared from bulk TT by mechanical dissociation, as previously described [14]. Briefly, frozen TT were thawed and washed with phosphate-buffered saline (PBS) and cut into small pieces (~ 2–4 mm) using a surgical scalpel. Cell dissociation was performed using gentleMACS dissociator (Miltenyi Biotec, Bergisch Gladbach, Germany) without enzymatic digestion. Cell suspension was then passed through a 100-µM cell strainer to remove cell aggregates and debris, and washed multiple times with PBS.

### Cell sorting

Single-cell suspensions from TT were resuspended in 100-µl flow cytometry staining buffer (PBS with 1% FCS and 0.1% sodium azide). Fc receptors (FcR) were blocked using FcR Blocking Reagent (Miltenyi Biotech, Bergisch Gladbach, Germany), and 7-AAD viability dye (eBioscience, San Diego, USA) was used to gate live cells. Cells were stained with cell surface antibodies against CD3-allophycocyanin-Cy7 (clone SK7, BD Pharmingen, San Jose, USA) and CD33-allophycocyanin (clone WM53, BD Pharmingen). Cells were washed twice with flow cytometry staining buffer and re-suspended in Pre-Sort buffer (BD Biosciences). BD FACSAria III SORP cell sorter on BD FACSDiva software (BD Biosciences) was used for sorting pure CD33^+^(7AAD^–^CD3^–^CD33^+^) populations. Relevant procedures were followed to ensure minimum sorter-induced cell stress (SICS), as previously described [15]. Flow cytometric representations were performed on FlowJo V10 software (FlowJo, Ashland, USA).

### RNA extraction and amplification

Total RNA was isolated from sorted, pure tumor-infiltrating CD33^+^ myeloid cells, from 13 CRC patients using RNA/DNA/protein purification Plus Micro Kit (Norgen Biotek Corporation, Ontario, Canada) according to the manufacture’s protocol. RNA was then amplified using 5X MessageAmp™ II aRNA Amplification Kit (Invitrogen) as per the manufacture’s protocol. RNA concentrations were determined using Qubit RNA HS or Broad Range Assay Kits (Invitrogen, California, USA).

### Quantitative real-time reverse transcriptase PCR (qRT-PCR)

Purified RNA (1 µg) was reversely transcribed to cDNA using QuantiTect Reverse Transcription Kit (Qiagen). PCR reactions for *ARG1*, *IDO1*, *IL1B*, *CCR2*, *CD274*, *LGALS9* and *β-actin* were performed using PowerUp SYBR Green Master Mix (Applied Biosystems) and QuantStudio 6/7 Flex Real-time PCR system (Applied Biosystems, California, USA). Relative gene expression was normalized to *β-actin* and calculated using 2^−ΔΔCT^ method. Additionally, the relative expression for each gene was calculated by normalizing the expression in CD33^+^ cells to data from sorted tumor-infiltrating CD8^+^ T cells from the same patients [16], after the initial normalization to *β-actin*. Primer sequences are presented in Table 2.

**Table 2:** Primer sequences for qRT-PCR

Primer	Sequence
*ARG1*	Forward, 5′- GTGGAAACTTGCATGGACAAC -3′ Reverse, 5′- AATCCTGGCACATCGGGAATC -3′
*IDO*	Forward, 5′- GCCAGCTTCGAGAAAGAGTTG -3′ Reverse, 5′- ATCCCAGAACTAGACGTGCAA -3′
*IL1B*	Forward, 5′- ATGATGGCTTATTACAGTGGCAA -3′ Reverse, 5′- GTCGGAGATTCGTAGCTGGA -3′
*IL6*	Forward, 5′- ACTCACCTCTTCAGAACGAATTG -3’ Reverse, 5′- CCATCTTTGGAAGGTTCAGGTTG -3′
*CCR2*	Forward, 5′- CCACATCTCGTTCTCGGTTTATC -3′ Reverse, 5′- CAGGGAGCACCGTAATCATAATC-3′
*CD274*	Forward, 5′- TGGCATTTGCTGAACGCATTT -3′ Reverse, 5′- TGCAGCCAGGTCTAATTGTTTT -3′
*LGALS9*	Forward, 5′- GGGCGCAGACAAAAACCTC -3′ Reverse, 5′- GGAGTAGAGAACATCTGTCCAGG -3′
*β-actin*	Forward, 5′- AGAGCTACGAGCTGCCTGAC -3′ Reverse, 5′- AGCACTGTGTTGGCGTACAG -3′

### Library preparation

cDNA libraries were generated amplified RNA using Exome TruSeq Stranded mRNA Library Prep Kit (illumina, San Diego, USA) according to the manufacturer’s protocol, and as previously described [17]. Quality passed libraries were subjected to clustering using TruSeq PE Cluster Kit v3-cBot-HS (illumina). The clustered samples were sequenced on an illumina HiSeq 4000 instrument using HiSeq 3000/4000 SBS kit (illumina).

### RNA-Sequencing data processing and analyses

RNA-Seq data were analyzed and illustrated using multiple bioinformatics tools. Pair end reads were quality-trimmed and aligned to the hg19 human reference genome in CLC Genomics Workbench 12 (Qiagen) [17]. The levels of expression of transcripts were measured as the score of TPM (Transcripts Per Million) mapped reads. Abundance data were subsequently subjected to differential gene expression analyses to compare transcriptome of tumor-infiltrating CD33^high^ myeloid cells from CRC patients with advanced stages versus early stages. The Z-scores were calculated from the TPM values as previously described [18]. Differential expression analyses were also performed to compare the transcriptomes of sorted tumor-infiltrating CD33^high^ myeloid cells with transcriptome of sorted tumor-infiltrating CD8^+^ T cells (used as controls) from the same patient cohort [16].

Volcano plots were generated using OriginPro 2020 software (OriginLab Corporation, Massachusetts, USA) with Log2 FC > 2 and *P* value cutoff < 0.05. The Gene Ontology (GO) analysis and Kyoto Encyclopedia of Genes and Genomes (KEGG) pathway enrichment analyses of differentially expressed genes (DEGs) were performed by the Database for Annotation, Visualization and Integrated Discovery (DAVID) tool, as previously described [19]. PCA plots and gene ontology clustering were performed using Integrated Differential Expression and Pathway (iDEP.91, South Dakota State University, USA) online analysis tool. Raw TPM values were uploaded and computed to identify and generate various illustrations for DEGs. K-Means clustering (iDEP.91) and STRING (https://string-db.org/, ELIXIR Core Data Resources) were used for performing gene enrichment and network enrichment analyses.

### Data alignment with the cancer genome atlas (TCGA) colorectal cancer

The top 100 upregulated genes and 100 downregulated genes from advanced stage vs. early stage comparison of CD33^+^ myeloid cells were selected for analysis in the TCGA CRC dataset. Of these 100 upregulated genes, 99 genes were annotated in the TCGA CRC RNA-Seq dataset, of which 55 genes (56%) had higher expression in patients with poorer disease-specific survival. For the 100 downregulated genes, 96 genes were annotated in the TCGA dataset, of which 42 genes (44%) had lower expression in patients with poorer disease-specific survival. The selected 55 upregulated and 42 downregulated genes were used as the ‘poorer prognosis CD33^+^ gene signature’ (ppCD33sig). The ppCD33sig score was calculated as the ratio of the average expression of the 55 upregulated genes to the average of the 42 downregulated genes. Therefore, ppCD33sig was identified from data of our cohort and its prognostic significance was confirmed from the TCGA datasets.

## Results

### Characterization of sorted tumor-infiltrating CD33^+^ myeloid cells in CRC patients

**Fig. 1:**
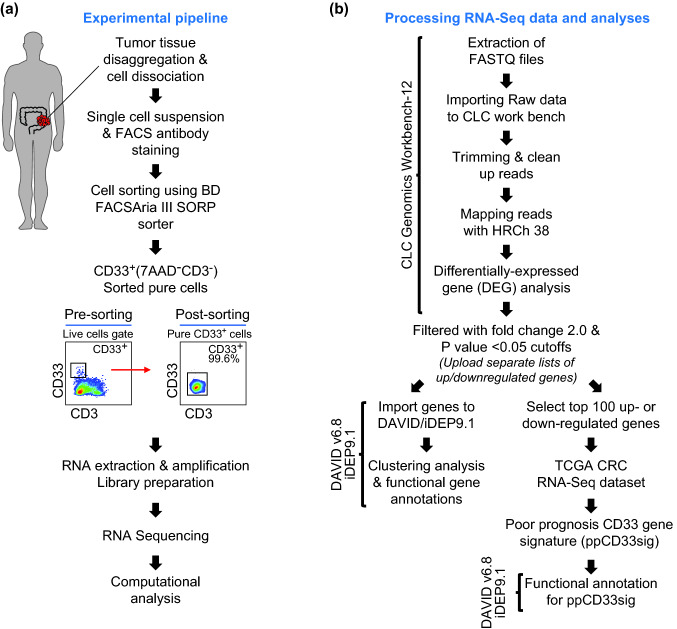
Schematic representation of study design. Tumor tissue specimens from 13 CRC patients were used to sort highly pure CD33^high^ myeloid cells. Libraries were generated from sorted cells for RNA-Seq. Different bioinformatics tools were utilized for analyses and visualization of RNA-Seq data. The experimental flowchart of this study is shown in (**a**), and the workflow for RNA-Seq data pre-processing and analysis using various bioinformatics tools is shown in (**b**)

CD33 is expressed on a heterogenous population of myeloid cells, and it does not distinguish MDSCs from TAMs or tumor-associated neutrophils (TANs) [20]. In addition, CD33 can be expressed on NK cells, but at lower levels compared to myeloid cell lineage [21, 22]. CD33^high^ populations predominantly comprise monocytic populations and include cells with potentially high suppressive characteristics as identified by several groups [23]. Therefore, we sorted CD33^high^ cells to avoid other cells expressing dim or low levels of CD33 from tumor tissues of CRC patients with varying disease stages to perform RNA-Seq. Data from RNA-Seq were analyzed using various bioinformatics tools. A schematic diagram of the overall study design is shown in Fig. 1.

**Fig. 2:**
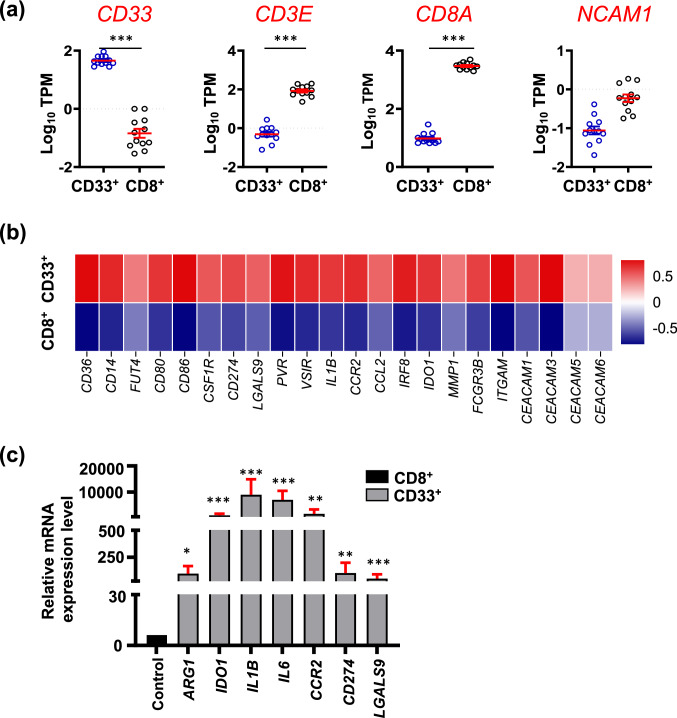
Transcriptome analyses confirming the phenotypic and functional characteristics of sorted CD33^+^ myeloid cells. Tumor tissues from CRC patients were used to sort highly pure CD33^+^ myeloid cells and CD8^+^ T cells (used as controls), and differential gene expression analyses were performed on RNA-Seq data. Scatter plots show gene expression levels as the mean of Log10 TPM ± standard error of the mean (SEM) for *CD33*, *CD3E*, *CD8A* and *NCAM1* (gene for CD56) genes (**a**). Heatmap represents the fold change in gene expression levels of myeloid phenotypic and functional markers in CD33^+^ vs. CD8^+^ cells as Z-scores, which were calculated from TPM values (**b**). Bar plot shows the mRNA expression levels of *ARG1*, *IDO*, *IL1B*, *IL6*, *CCR2*, *CD274* and *LGALS9* in CD33^+^ cells normalized to CD8^+^ cells (used as controls), analyzed by qRT-PCR. The relative expression of each gene was calculated by normalization with *β-actin*. Mean + standard error of the mean (SEM) is shown in red (**c**). Statistical significance was calculated using non-parametric paired *t*-test; *P < 0.05; ***P < 0.001

RNA-Seq data analyses enabled us to confirm the purity of sorted populations by comparing transcriptomes of sorted tumor-infiltrating CD33^high^ cells with those of CD8^+^ T cells from the same patients, and determine the gene expression levels of phenotypic markers for myeloid and lymphoid cell populations (Fig. 2a). We found that sorted tumor-infiltrating CD33^high^ cells expressed high levels of *CD33* gene, while lacked gene expression of *CD3E, CD8A* and *NCAM1* (gene for CD56), compared to CD8^+^ T cells (Fig. 2a). Next, we investigated gene expression levels of myeloid phenotypical and functional markers within CD33^high^ cells to investigate the heterogeneous populations, which constitute these cells. We found that tumor-infiltrating CD33^high^ cells showed high gene expression levels of *CD36, CD14, FUT4* (CD15)*, CD80, CD86, CSF1R* and immune checkpoint ligand genes including *CD274* (PD-L1)*, LGALS9* (galectin-9)*, PVR* and *VSIR* (Fig. 2b). Moreover, *IL1B, IDO1, MMP1* and *CAECAM1/3/5/6* were also upregulated in CD33^high^ cells (Fig. 2b).

Next, we performed qRT-PCR to investigate and validate mRNA expression levels of some key genes related to functional activity/recruitment or activation of myeloid cells including *ARG1*, *IDO1*, *IL1B*, *CCR2*, *CD274* (PD-L1), *LGALS9* (galectin-9) (Fig. 2c). We found all of these genes were expressed at significantly higher levels in CD33^high^ than CD8^+^ T cells in the TME (Fig. 2c). Together, these data confirm the purity and functional activity of the sorted tumor-infiltrating CD33^high^ cells.

### Transcriptomes and functional annotations of differentially expressed genes in tumor-infiltrating CD33^+^ myeloid cells in CRC patients with early and advanced stages

**Fig. 3:**
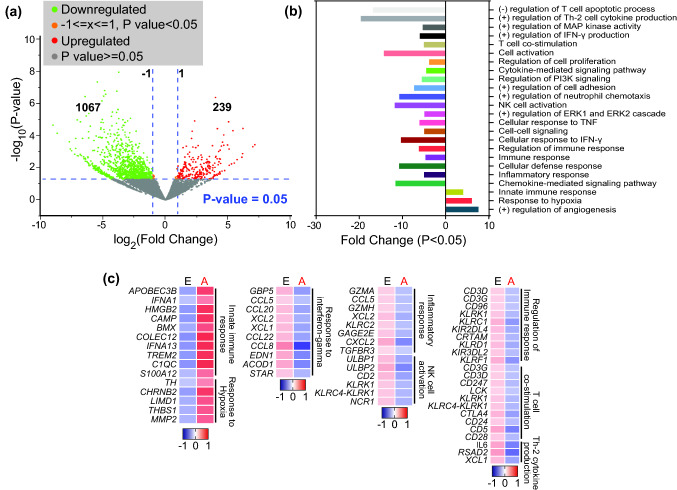
Transcriptome analyses of differentially expressed genes in tumor-infiltrating CD33^+^ myeloid cells in CRC patients with early and advanced stage. We divided the patients into two groups based on disease stage; early stage (stages I and II, *n* = 8) and advanced stage (stages III and IV, *n* = 5), to compare their transcriptomic data. Volcano plot shows overall differentially regulated genes in patients with advanced stage compared to patients with early stage; red dots represent upregulated, green dots represent downregulated and gray dots represent genes, which remained unchanged with *P* values as a function of fold change and indicate the vertical dotted line to delimit > twofold change and *P* < 0.05 cutoffs (**a**). Bar plot shows functional annotations for the genes that were significantly upregulated or downregulated in CD33^+^ myeloid cells from advanced vs. early stages (**b**). Heatmaps show the Z-scores for various up- or downregulated genes and associated pathways in advanced vs. early stages (**c**)

For differential gene expression analyses, we divided patients into two groups based on disease stage; early stage (stages I and II, *n* = 8) and advanced stage (stages III and IV, *n* = 5). We found 1,306 differentially expressed genes (DEGs) between tumor-infiltrating CD33^+^ cells in CRC patients with early and advanced stages (Log2 FC ≥ 2 and *P* value cutoff ≤ 0.05, Supplementary Table 1). 239 genes were upregulated, while 1067 genes were downregulated in advanced stages (Fig. 3a). Hierarchical gene clustering across all patients is shown in Supplementary Fig. 1a.

To identify the functional pathways of DEGs from advanced versus early stage comparison, we performed gene ontology (BP) and KEGG pathway analyses using DAVID platform. We found that innate immune response-, response to hypoxia- and angiogenesis-related genes were significantly upregulated in advanced stages (*P* ≤ 0.05, Fig. 3b, c). On the other hand, genes related to inflammatory response, T cell/NK cell activation, response to IFN-γ, and chemokine-mediated signaling were significantly downregulated in advanced stages, compared with early stages (*P* ≤ 0.05, Fig. 3b, c). These data suggest that adaptive anti-tumor immune responses could be compromised in advanced disease stages due to impaired innate immune responses initiated by exerted by myeloid cells.

We also generated enrichment networks of the downregulated pathways to identify possible functional interactions in patients with advanced-stage compared to early-stage CRC (Supplementary Fig. 1b). We found that cell migration/chemotaxis, chemokine-mediated signaling and immune response were downregulated in patients with advanced-stage disease (Supplementary Fig. 1b). These data further confirm our functional pathways enrichment in CD33^+^ TILs from advanced stages of CRC.

### Analyses of functional pathways enriched in tumor-infiltrating myeloid cells from various stages of CRC

**Fig. 4:**
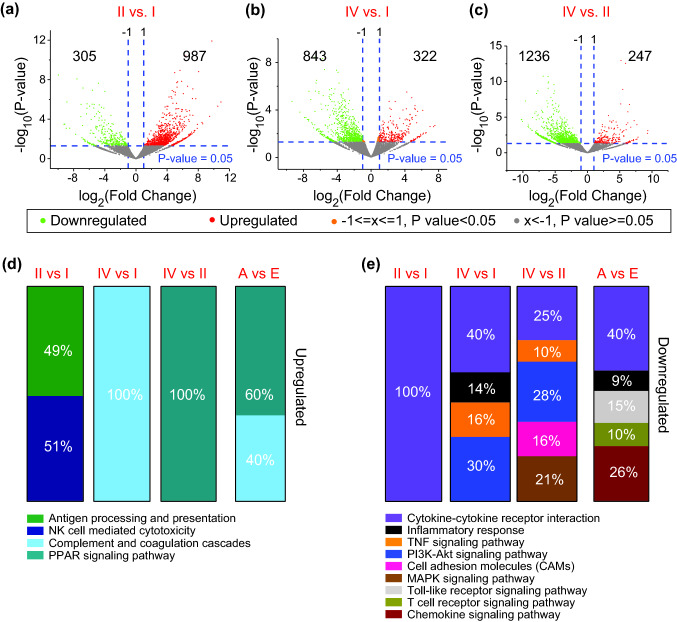
Functional pathway analyses of deregulated genes from stage-wise comparison. Differentially expressed genes from stage-wise comparisons were analyzed using the DAVID platform to identify functional pathway enrichments. Volcano plots show the deregulated genes obtained from stage II vs. I (**a**), IV vs. I (**b**), and IV vs. II (**c**). Bar charts show significantly upregulated (**d**) and downregulated (**e**) KEGG pathways enriched in each comparison, accompanied with the percentage of enriched genes in each pathway

Next, we identified DEGs from stage-wise comparisons and subsequently investigated upregulated/downregulated pathways (Fig. 4a–c and Supplementary Table 1). We found that antigen processing and presentation- and NK cell-mediated cytotoxicity-related genes were enriched in stage II compared to stage I (Fig. 4d). Complement and coagulation cascade-related genes were upregulated in stage IV compared to stage I (Fig. 4d). This enrichment was also evident in advanced (A) vs early (E) stage comparisons (Fig. 4d). Similarly, enrichment of PPAR signaling-related genes in stage IV compared to stage II was also evident in advanced vs early comparison (Fig. 4d).

In contrast, we found that the majority of pathways were in the downregulated gene panel in stage comparisons. Cytokine–cytokine receptor interaction-related genes were significantly downregulated in all comparisons (Fig. 4e). In addition, inflammatory response-, TNF signaling- and PI3K–Akt signaling-related genes were also diminished in stage IV versus I (Fig. 4e); while PI3K–Akt signaling-, cell adhesion molecules-, MAPK- and TNF signaling-related genes were also downregulated in stage IV vs II (Fig. 4e). Overall, cytokine–cytokine receptor interaction-, chemokine signaling-, Toll-like receptor signaling-, inflammatory response- and TCR signaling-related genes were significantly downregulated in advanced vs early stages (Fig. 4e). These results implicate that innate immune responses involved in T cell activation and immune cell recruitment could be compromised in advanced disease stages.

### Analyses of steadily upregulated and downregulated genes in CRC patients with varying disease stages

Next, we investigated genes and their associated pathways, which were steadily upregulated (Supplementary Fig. 2a) or downregulated in tumor-infiltrating CD33^+^ myeloid cells of CRC patients across disease stages (Stages I, II and IV) (Supplementary Fig. 2b). We found that particular genes were steadily upregulated (*n* = 33) and downregulated (*n* = 267) across the stages. Genes such as human leukocyte antigen (HLA) complex genes *HLA-DQA2/HLA-DQB2* encoding HLA-Class II molecules [24], cancer/testis (CT) antigen *XAGE1B* [25] and *FOLR* genes, which are related to multiple cancers [26], were steadily upregulated in advanced stage. In contrast, several genes including interleukin-22 (*IL22*), epidermal growth factor receptor (*EGFR*), and *IL6* were downregulated steadily with disease progression. These data implicate that tumor-infiltrating CD33^+^ myeloid cells in advanced stages could have impaired activation and limited ability of cell proliferation.

### Aligning DEGs with TCGA colorectal cancer reveals a gene signature of poor survival

The deregulated genes in CD33^+^ cells in advanced disease stage were used to calculate the poor prognosis CD33^+^ gene signature (ppCD33sig) score as described in the method section and shown in Supplementary Table 2. The CRC TCGA cases were labeled as high, intermediate and low groups according to the ppCD33sig.

**Fig. 5:**
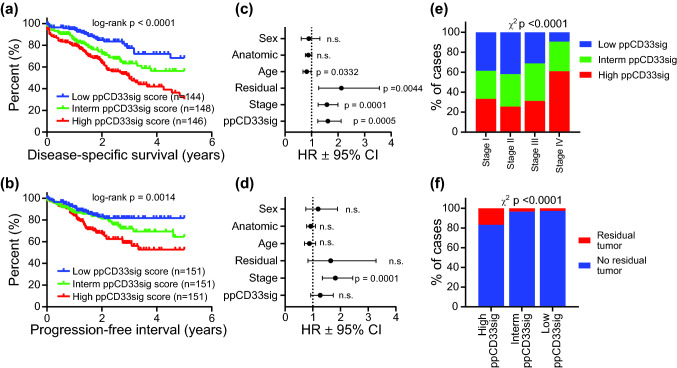
The prognostic significance of ‘poor prognosis CD33^+^ gene signature’ (ppCD33sig) in the TCGA CRC dataset. Selected genes were used to determine the ppCD33sig score, calculated as the ratio of the average expression of the 55 upregulated genes to the average of the 42 downregulated genes. The ppCD33sig was evaluated in the TGCA CRC RNA-Seq dataset. Disease-specific survival (**a**) and progression-free interval (**b**) were compared between patients with high (top 33%), intermediate (middle 33%) or low (bottom 33%) ppCD33sig scores. The number (*n*) of patients in each of ppCD33sig groups and the Log-rank *P* value from Mantel–Cox test (GraphPad Prism v8.4) are indicated. Multivariate analyses using Cox proportional-hazard model (MedCalculator v12.7) comparing the ppCD33sig (high, interm, low), disease stage (Stages IV, III, II, I), residual disease (yes, no), age (< 55, 55–64, 65–74, > 74 years of age), anatomic locations (7 different locations), and sex (male, female) for disease-specific survival (DSS) (**c**) and progression-free interval (PFI) (**d**). Data shown are the hazard ratio (HR) ± 95% confidence interval (CI) and the multivariate *P* values are indicted (n.s.: not significant) (**c**,** d**). Distribution of patients with high, intermediate, or low ppCD33sig scores across disease stages (**e**). The presence of residual disease in patients with different ppCD33sig scores (**f**). Stated *P* values are from Chi-square (χ^2^) test was used

Patients with high ppCD33sig score had poorer disease-specific survival (DSS) and shorter progression-free interval (PFI) compared to patients with intermediate or low ppCD33sig score (Fig. 5a, b). The ppCD33sig was an independent prognostic indicator for DSS (Fig. 5c) but not PFI (Fig. 5d), as indicated by multivariate analysis using Cox proportional-hazard model even in the presence of disease stage as another indicator. Patients at advanced stages (III and IV) were more likely to have high ppCD33sig score (Fig. 5e). Patients with high ppCD33sig score were more likely to have residual disease after primary therapy (Fig. 5f). The splenic flexure, descending and sigmoid colon anatomical locations (left-sided colon cancer) were more likely to have a high ppCD33sig score whereas the transverse colon and the right-sided colon cancer (hepatic flexure, ascending colon and the cecum) were more likely to have a low ppCD33sig score (Supplementary Fig. 3a). The ppCD33sig score did not differ between males and female (Supplementary Fig. 3b) or age groups (Supplementary Fig. 3c). Next, we classified the patient cohort into two groups; high ppCD33sig score and low ppCD33sig score. We found that 6 patients with low and 7 patients with high ppCD33sig score (Supplementary Table 3). Hierarchical clustering showed 372 upregulated and 1991 downregulated genes with Log2 FC > 2 and *P* value ≤ 0.05, when comparing high score with low score patients (Supplementary Fig. 3d). Moreover, our gene ontology-biological process analyses showed that innate immune response/myeloid differentiation-related genes were upregulated, while adaptive immune response/cell differentiation/migration-related genes were downregulated in high score, compared with low ppCD33sig score patients (Supplementary Fig. 3e and Supplementary Table 4). Additionally, protein–protein interaction (PPI) and enrichment network analyses revealed that innate immune responses-related genes, which were upregulated in patients with high ppCD33sig score, formed a significant network with *P* value of < 1.0E-16 (Supplementary Fig. 4a). In contrast, adaptive immune responses related genes, which were downregulated genes in high ppCD33sig score also formed a significant network with *P* value of < 1.0E-16 (Supplementary Fig. 4b). These data further confirm our findings that in CD33^+^ cells, innate immune responses were upregulated and adaptive responses were downregulated in advanced stages of CRC, compared with early stages.

## Discussion

Transcriptomic profiling of myeloid cells has been previously carried out in different cancers [27]. However, it is of particularly higher significance in inflammation-related cancers, such as CRC, because of changes in the balance between different immune cell populations at the site of inflammation. Myeloid cells are the most common mediators of inflammation [28], however, some subsets of myeloid cells can act as potent immunosuppressive cells and have been detected in the CRC TME with high frequencies. We have previously reported the expansion of different MDSC subsets in the TME of CRC patients [11]. More recently, we reported the different epigenetic regulations and other molecular pathways in colorectal tumor-infiltrating MDSC subsets within TME, which could contribute to their expansion and functionality [19]. In the present study, we investigated the transcriptome of tumor-infiltrating myeloid cells to uncover molecular pathways, which are upregulated or downregulated in CRC patients as disease progresses from early to advanced stages.

Myeloid cell populations within the TME are plastic and heterogeneous with various phenotypical and molecular characteristics; the switch of tumor-infiltrating myeloid cells from a “pro-inflammatory” to “anti-inflammatory or immunosuppressive” can be triggered by multiple mechanisms driven by tumor-induced factors, T regulatory cells or metabolic pathways [8, 29]. Dynamic interactions between different myeloid cell subsets and T cells within the TME are one of the key factors which dictate tumor progression [30]. Anti-tumor immune responses require a shift in the balance between immunosuppressive cells and potent T cell activation in favor of effective anti-tumor immunity and a pro-inflammatory environment instead of an immunosuppressive one [31].

CD33^+^ populations within the TME are enriched for HLA-DR^–^ populations and comprise different MDSC subsets, including CD33^+^HLA-DR^–^CD14^+^CD15^–^ monocytic MDSCs, CD33^+^CD14^+^CD15^–^ polymorphonuclear MDSCs and CD33^+^HLA-DR^–^CD14^–^CD15^–^ immature or early-stage MDSCs [32]. Since we did not sort HLA-DR^−^ populations, the sorted cells comprise of a heterogeneous population of myeloid cell lineage, which primarily could include immunosuppressive subsets of myeloid cells, such as MDSC and TAMs, but also include some HLA-DR-expressing APCs. The presence of these populations was evident in the transcriptome of sorted cells, since genes encoding CD14, CD15 (*FUT4*), and other myeloid cell phenotypic markers such as CD36, CD80 and CD86 were higher in sorted cells. In addition, the immunosuppressive potentials of CD33^high^ populations was confirmed by expressing high levels of immunosuppressive factors, such as arginase-1 (ARG1) and indoleamine-pyrrole 2,3-dioxygenase (IDO), inhibitory immune checkpoint ligands, such as PD-L1 and galectin-9, and cytokines important for MDSC recruitment and function/activation, such as CCR2, IL-1β and IL-6 [33].

CD33^high^ populations comprise of diverse monocytic cell populations involved in lymphocyte recruitment and also in immunosuppression [7]. Moreover, the presence of CD33^high^ immunosuppressive monocytes has been recorded in various cancers [34, 35]. We found that genes related to numerous vital immune modulatory pathways, including the regulation of T cell/NK cell activation and response to IFN-γ, were downregulated in myeloid cells from patients with advanced disease stage, compared to those with early stages. These results implicate that myeloid cells in advanced stage disease could lose their pro-inflammatory properties and acquire pro-tumorigenic properties promoting the suppression of anti-tumor immunity, primarily T cell and NK cell activation, and supporting tumor progression. NK cells are considered as primary innate immune cells with anti-tumor activity against non-MHC-expressing tumor cells [36]. Here, we found that genes related to NK cell activation were downregulated in tumor-infiltrating myeloid cells from advanced stage patients, suggesting the limited activity of NK cells in progressive disease stages.

Importantly, we found that genes related to innate immune responses were upregulated, while genes related to adaptive immune responses were downregulated in tumor-infiltrating myeloid cells from patients with advanced stage. M2 macrophages, TAMs and MDSC constitute innate immune cells, which augment tumor growth, invasion and metastasis, and have been previously associated with aggressive CRC tumors [37]. Moreover, it has been reported that increased levels of suppressive myeloid cells in the TME could be responsible for regulating adaptive immune responses [38]. In addition, we found that cell chemotaxis-, myeloid leukocyte migration-, and chemokine-mediated signaling-related genes were downregulated in patients with advanced stage. Immune cell migration is critical in prompting immune responses and dictates outcomes of different pathological conditions, especially inflammation-related cancers such as CRC [39]. These data show that inactivation/impaired adaptive immune responses could play a vital role in CRC disease progression.

Myeloid suppressor cells can support tumor growth by favoring angiogenesis, suppressing immune responses and promoting metastasis [40]. Our data provide evidence that *MMP9*, known to be associated with the ‘angiogenic switch’ [41] was upregulated in patients with advanced stage. In concordance with these evidences, genes related to the positive regulation of angiogenesis and response to hypoxia were also upregulated in tumor-infiltrating myeloid cells from patients with advanced stage, potentially suggesting that hypoxia can switch on genes in tumor-infiltrating myeloid cells to drive angiogenesis and consequently metastasis in patients with advanced disease stage. Hypoxia occurs in most growing solid tumors as a result of increased oxygen consumption by growing tumor cells and has been associated with tumor aggressiveness and resistance to therapy [42]. Additionally, hypoxia can affect the plasticity and heterogeneity of tumor cells, alter the phenotypic states of immune cells, including myeloid cells, and induce several pathways that support tumor growth and progression, such as angiogenesis [29, 42]. Our results suggest a plausible link between hypoxia and disease progression via myeloid cells with potential immunosuppressive properties. Moreover, we found some CT antigen-related genes were upregulated in tumor-infiltrating myeloid cells in advanced stages. Certain CT antigens have been previously identified as prognostic biomarkers for CRC progression [43]. Our data show that CT antigens may be expressed on tumor-infiltrating myeloid cells and could be explored as biomarkers for CRC progression.

It is vital to note that while our results provided evidence for the regulation of various genes in tumor-infiltrating myeloid cells in CRC patients with different disease stages, the affected pathways and the functional significance of these data require further investigations to validate these findings. Our data indicate various pathways by which myeloid cells could contribute to the progression of CRC. In an attempt to decipher the clinical relevance of our data, we aligned the top upregulated/downregulated genes with the TCGA CRC RNA-Seq data, and identified “the poorer prognosis CD33^+^ gene signature”. We found that this gene signature is an independent prognostic indicator for DSS (timeframe of diagnosis until death), but not PFI (survival timeframe without worsened disease outcomes). These results suggest the potential specificity of the gene signature identified to survival rates, without having an impact on disease progression.

Based on TCGA analyses, we also showed that patients at advanced stages were more likely to have high ppCD33sig score, and patients with high ppCD33sig score were more likely to have residual disease after primary therapy. These findings implicate the potential link between high ppCD33sig score, tumor recurrence and tumor resistance to therapy. In line with this, several reports have demonstrated the clinical significance of colorectal tumor-infiltrating myeloid cells in dictating tumor relapse and influencing the response to therapy [44]. Cytokines and chemokines within the TME are crucial soluble factors which determine the nature of the TME (i.e., suppressive or pro-inflammatory) and can orchestrate the function of immune cells and regulate their migration and accumulation at tumor sites [45]. Coincidently, we found that innate immune response-related genes, such as cytokines and chemokines, were significantly upregulated in high ppCD33sig score; these chemokines and cytokines are more likely to be involved with the chemotaxis, differentiation and survival of tumor cells and immunosuppressive cells, including Tregs, TAMs and MDSCs [8, 10]. On the other hand, genes associated with the activation of adaptive immune response were significantly downregulated in CRC patients with high ppCD33sig score, indicating the suppressive potential of tumor- infiltrating myeloid cells in these patients. These findings further support our previous results obtained by comparing the transcriptomes of tumor-infiltrating myeloid cells from advanced vs. early stages.

Overall, this study provides critical insights into the pathways affected in tumor-infiltrating myeloid cells during the progression of CRC and their potential contribution to disease progression, and identifies a CD33^+^ gene signature which predicts poor disease-specific survival in CRC patients. However, further validations are required for the functional attributes of the identified gene signature in myeloid cells.

## Availability of data and material

The datasets used and/or analyzed during the current study are available from the corresponding author on request.

### Electronic supplementary material

Below is the link to the electronic supplementary material.

Supplementary file1 (PDF 4153 kb)

Supplementary file2 (XLSX 307 kb)

Supplementary file3 (XLSX 479 kb)

Supplementary file4 (XLSX 21 kb)

Supplementary file5 (XLSX 26 kb)

## Abbreviations

CRC: Colorectal cancer
DAVID: Database for annotation, visualization and integrated discovery
DSS: Disease-specific survival
DEGs: Differentially expressed genes
ICI: Immune checkpoint inhibitor
MDSC: Myeloid-derived suppressor cell
PCA: Principle component analysis
PFI: Progression-free interval
ppCD33sig: Poor prognosis CD33 gene signature
PPI: Protein–protein interaction
TAM: Tumor-associated macrophage
TAN: Tumor-associated neutrophil
TCGA: The cancer genome atlas
Treg: T regulatory cell
