# Supplementary material for: Differential gene expression of tumor-infiltrating CD33+ myeloid cells in advanced- versus early-stage colorectal cancer
Source: Cancer Immunol Immunother. 2020 Sep 30;70(3):803–15. doi: 10.1007/s00262-020-02727-0 (PMC7906947; doi:10.1007/s00262-020-02727-0)
Supplement: Supplementary file 1 — Supplementary file1 (PDF 4153 kb) [file 262_2020_2727_MOESM1_ESM.pdf]

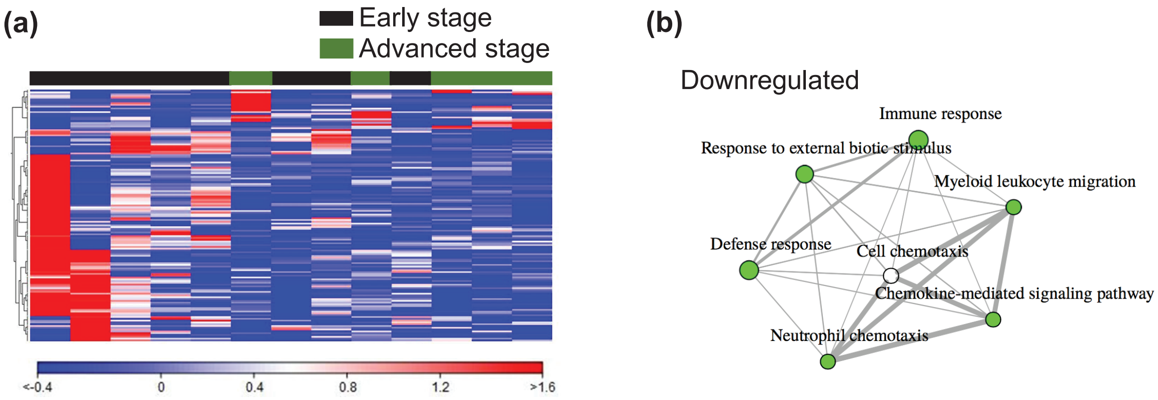

Supplementary Figure 1. Differential gene expression analyses of tumor-infiltrating CD33+ cells in patients with early and advanced disease stages. Hierarchical clustering shows differentially expressed genes between tumor-infiltrating CD33+ cells in patients with early and advanced stages (FC ≥2 and P value cutoff ≤0.05 **(a)**. Network analyses of pathways illustrated; the green dots represent downregulated pathways in tumor-infiltrating CD33+ myeloid cells in CRC patients with advanced compared to early stage disease **(b)**.

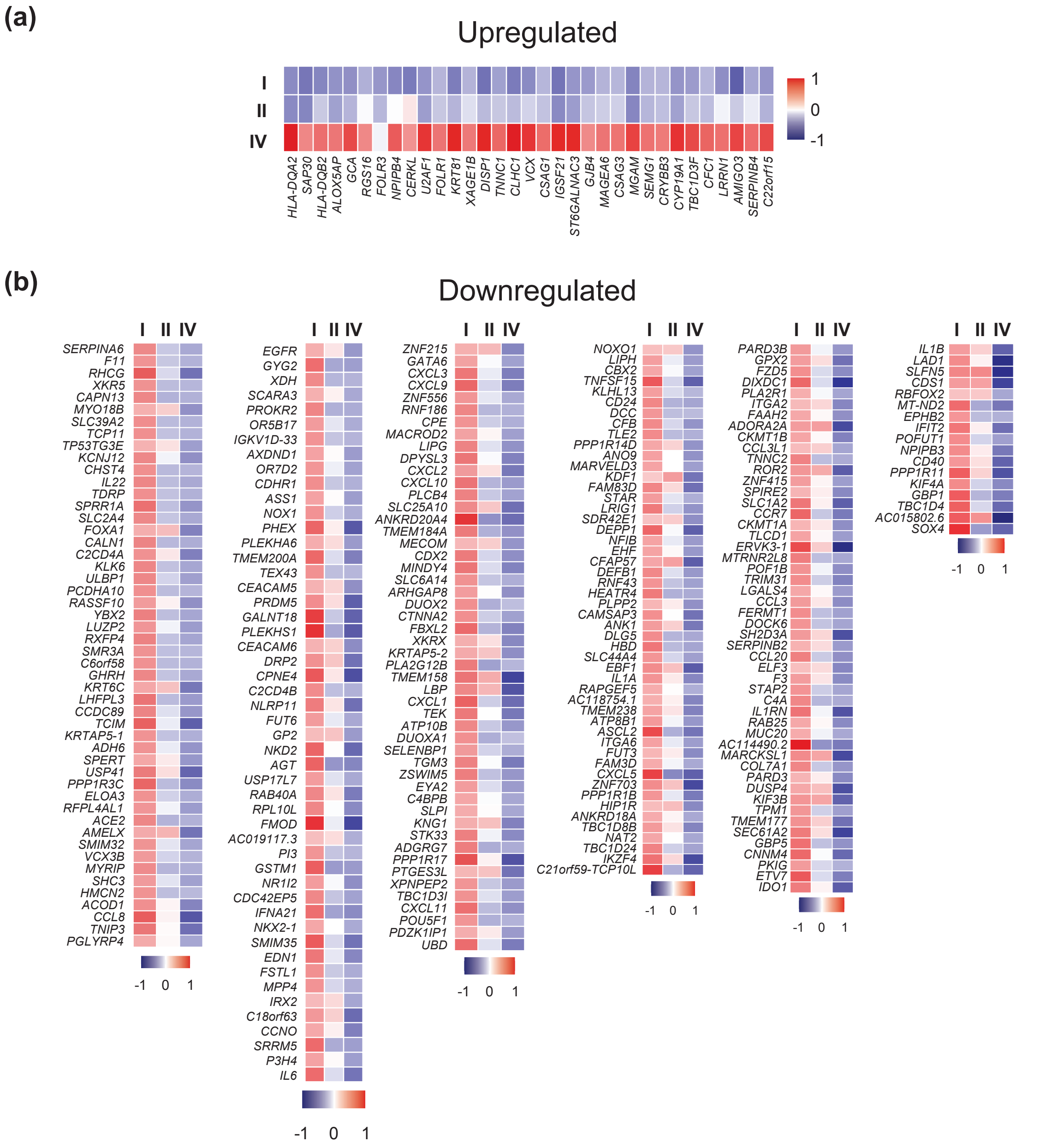

Supplementary Figure 2. Analyses of steadily deregulated genes in patients with the progression of CRC. Heatmaps show differentially expressed genes (Z-scores), upregulated **(a)** and downregulated **(b)**, in tumor-infiltrating $CD33^+$ myeloid cells.

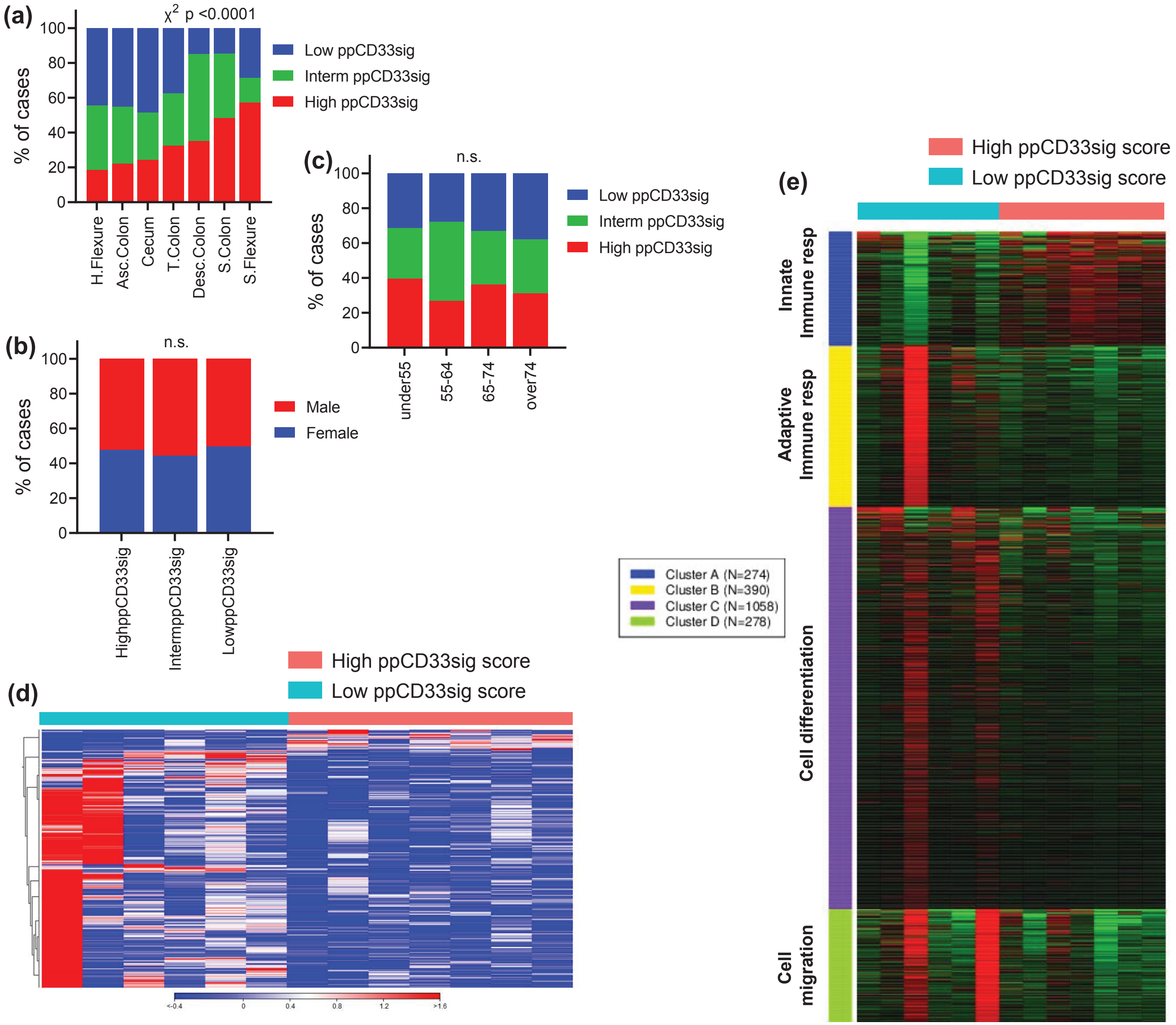

Supplementary Figure 3. Supporting data for the evaluation of the poor prognosis CD33⁺ gene signature in the TCGA CRC dataset. Distribution of patients with high, intermediate, or low ppCD33sig scores across the different anatomical location of their colon cancer. Left-sided colon cancer patients (splenic flexure, descending and sigmoid colon) had higher ppCD33sig than patients with transverse colon and right-sided colon cancers (hepatic flexure, ascending colon and the cecum) by using Chi-square ($\chi 2$) test **(a)**. The ppCD33sig scores did not associate with sex **(b)** or patient age **(c)**. Chi-square test (n.s.: not significant). The ppCD33sig score for the 13 patients was calculated based on ratios between the average expression (TPM) of upregulated genes to the average expression of downregulated genes. Patients were dichotomized as high score (above median score) and low score (below median score) groups. Differential expression analyses were performed on data comparing high score vs. low score patients to identify deregulated and functional networks using iDEP platform. Hierarchical heatmap shows the distinct cluster of high score and low score patient groups (FC >2 and *P* value <0.05) **(d)**. Heatmap shows the four functional clusters from gene ontology enrichment analyses **(e)**.

(a)

Upregulated

GO-term	Description	FDR
GO:0045087	Innate immune response	3.59E-38

Number of nodes: 40
Number of edges: 50
Avg. node degree: 2.75
Avg. local clustering coefficient: 0.39
PPI enrichment *P* value: <1.0E-16

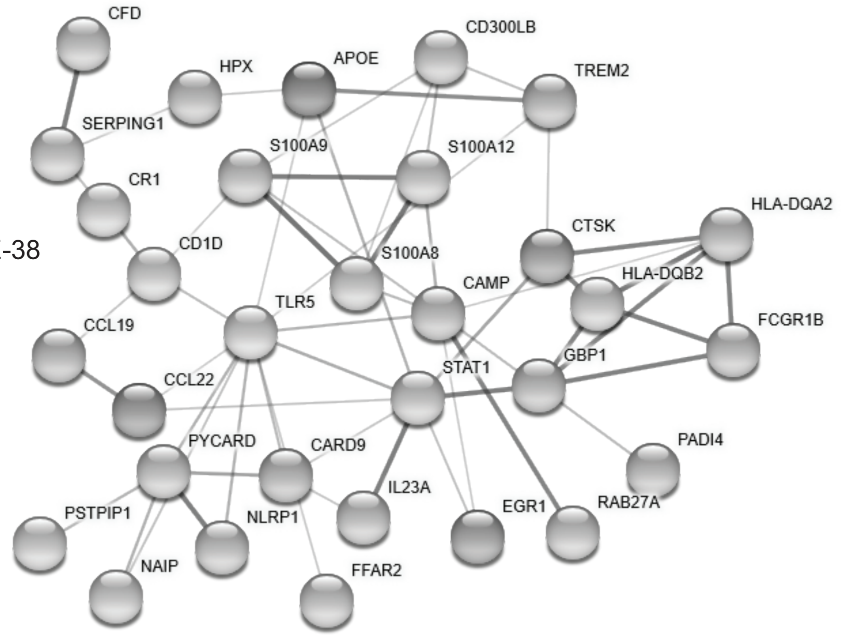

(b)

Downregulated

GO-term	Description	FDR
GO:002250	Adaptive immune response	4.65E-35

Number of nodes: 31
Number of edges: 124
Avg. node degree: 8
Avg. local clustering coefficient: 0.71
PPI enrichment *P* value: <1.0E-16

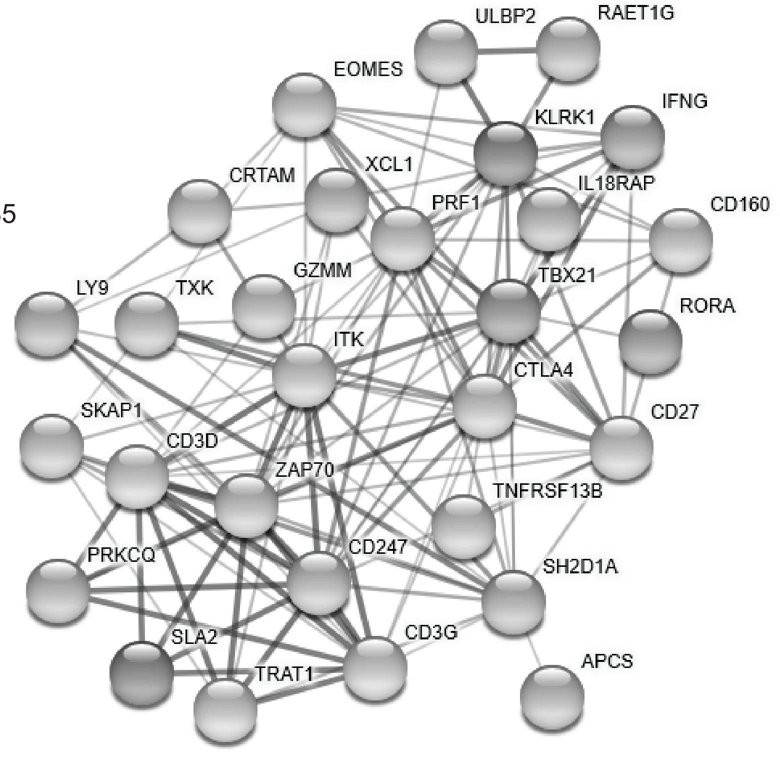

Supplementary Figure 4. PPI network analyses of deregulated genes in high ppCD33sig patients. The ppCD33sig score for the 13 patients was calculated based on the ratios between the average expression (TPM) of upregulated genes to the average expression of downregulated genes. PPI network analyses using the STRING database of significantly upregulated **(a)** and downregulated **(b)** genes obtained from analyses of high ppCD33sig vs. low ppCD33sig groups. The unconnected nodes were removed from the networks. Gene Ontology (biological process) description and false discovery rate (FDR) using whole transcriptome as reference are stated for each subnetwork. The overall network statistics are shown in the grey boxes.
